# Profiling of Primary Metabolites and Volatile Determinants in Mahlab Cherry (*Prunus mahaleb* L.) Seeds in the Context of Its Different Varieties and Roasting as Analyzed Using Chemometric Tools

**DOI:** 10.3390/foods10040728

**Published:** 2021-03-30

**Authors:** Mohamed A. Farag, Amira R. Khattab, Samir Shamma, Sherif M. Afifi

**Affiliations:** 1 Pharmacognosy Department, College of Pharmacy, Cairo University, Kasr El Aini St., Cairo 11562, Egypt; 2 Chemistry Department, School of Sciences & Engineering, The American University in Cairo, New Cairo 11835, Egypt; 3 Pharmacognosy Department, College of Pharmacy, Arab Academy for Science, Technology and Maritime Transport, Alexandria 1029, Egypt; Dr_amira_khattab@aast.edu; 4 Institute of Global Health and Human Ecology, School of Sciences and Engineering, The American University in Cairo, P.O. Box 74, New Cairo 11835, Egypt; samirnabhan@aucegypt.edu; 5 Pharmacognosy Department, Faculty of Pharmacy, University of Sadat City, Sadat City 32897, Egypt; shshsh38@hotmail.com

**Keywords:** solid-phase microextraction (SPME), metabolomics, *Prunus mahaleb* L., white and red mahlab, GC–MS, volatile profiling, roasting

## Abstract

Mahlab cherry (*Prunus mahaleb* L.) is a plant native to the Mediterranean basin and Eastern Europe, with several health benefits and culinary uses. We explored the compositional heterogeneity in the aroma profile and nutrients of three *P. mahaleb* seeds in the context of its cultivar type, i.e., white and red, and in response to roasting. A holistic untargeted metabolomics approach was employed for the first time using solid-phase microextraction (SPME–GC–MS) profiles of seed volatiles and primary metabolites coupled with chemometrics. Around 65 peaks belonging to sugars, fatty acids, esters and organic acids were identified by GC–MS. White mahlab from Egypt is rich in fatty acids, e.g., oleic and *α*-linolenic acids. Some acyl esters, e.g., glycerylmonostearate and *n*-butylcaprylate, characterized mahlab cultivars from various origins. A total of 135 volatiles were identified, with organic acids and aldehydes the most abundant. Aldehydes were the most discriminatory in seed origin and in accounting for its distinct aroma. Several roasting indices were identified, viz. 1-octanol, *γ*-caprolactone and isomintlactone. A direct relationship between furans and fatty acids was rationalized by cyclic transformation of the latter into furan derivatives. This study provides the first chemical evidence supporting the nutritional and flavor determinants of mahlab seeds, suggesting novel uses as a functional food.

## 1. Introduction

Mahlab or mahaleb cherry tree (*Prunus mahaleb* L. a member of family Rosaceae), also known as St. Lucie cherry, is found wild throughout the Mediterranean zone, as well as Eastern Europe and West Asia. It is cultivated for producing a highly valued global spice derived from the seeds contained within the cherry stones that is used to season sweet confections in Eastern Mediterranean countries. The powdered mahlab seed is used in small quantities to flavor food in Greece, Armenia and Turkey [1].

In Sudan, crushed kernels of white mahlab are used to manufacture traditional fragrances and for nourishing hair lotions in wedding preparations. In addition, consumption of soaked white mahlab seed is considered a remedy for the treatment of diarrhea in children [2]. The kernel oil has been exploited in specialty wines and liqueur, owing to its unique aroma. The kernel’s flavor is comparable to that of a bitter almond taste, particularly after chewing, with a pleasant aroma widely used in small quantities to flavor foods, such as bagels, pastries, biscuits, cupcakes, candies and cookies. Mahlab kernel is considered as a vital source of protein (28%) and carbohydrates (14%). In addition, its oil (31%) is incorporated in the manufacture of varnishes, specialty wines and lacquers. Traditionally, mahlab (St. Lucie cherry) kernels were used as tonics, diuretics, digestive aids, sedatives and antidiabetics [2]. Mahlab cherry exhibits anti-inflammatory and antioxidant activities, as well as antifungal and antibacterial effects, with decoctions made from different parts of the mahlab tree used locally for the treatment of asthma and the common cold [3].

The pleasant aroma and medicinal value of mahlab seed is attributed to its abundance in coumarins, such as herniarin and dihydrocoumarin, in addition to tannins and traces of cyanogenic glycosides [2]. Shams (2006) isolated a novel compound, namely 2-glucosyloxy-4-methoxy methyl *trans* cinnamate, from mahlab kernel, with seeds found rich in phenolic acids, e.g., *o*-coumaric acid glucosides, in addition to flavanols, e.g., quercetin-3-*O*-glucoside [4]. Shams and Schmidt (2007) reported that mahlab seed oil is dominated by linoleic, α-eleostearic and oleic acid in addition to tocopherols, i.e., γ-tocopherol, also accounting for its antioxidant effects [5]. The volatile composition derived from mahlab honey revealed high vomifoliol and coumarin levels likely derived from the plant itself [6]. With regard to differences among mahlab cultivars, white mahlab (*P*. *mahaleb*) seeds are richer in oil and protein and, hence, are preferred compared to black mahlab (*Monechma ciliatum*) seeds [2]; other differences in its metabolite composition have been suggested but are yet to be fully elucidated. Concerning the genus *Prunus*, this is the first metabolomics approach to address its different varieties, in addition to the impact of roasting. Our aim is to assess the compositional heterogeneity in the aroma profile and nutrients of *Prunus mahaleb* seeds in the context of its cultivar type and in response to roasting.

Analysis of food and spices has lately started to implement modern analytical approaches, e.g., metabolomics, where samples are examined in a rather untargeted, comprehensive manner. GC–MS analysis has been extensively adopted for metabolomics profiling in fruits and seeds to characterize their metabolite components, e.g., volatiles and non-volatiles [7]. Unlike common thermal methods for volatile extraction, e.g., distillation, solid-phase microextraction (SPME) is a convenient analytical technique that is solvent-free and requires minimal sample preparation for the enhancement of volatile recovery from gas or liquid samples throughout a fused-silica fiber accompanied by the eventual desorption of these analytes [8]. To better interpret such huge datasets, multivariate data analyses are often used, e.g., principal component analysis (PCA), in addition to supervised methods, viz., orthogonal projection to least squares discriminant analysis (OPLS-DA), which can simplify metabolite data complexity and ease sample classification.

To the best of our knowledge, this is the first report of the determination of the quality characteristics of *Prunus mahaleb* seeds, i.e., aroma and nutrient metabolites, in the context of different cultivars and post-roasting using GC–MS, assisted by multivariate data analysis tools. For aroma collection, headspace SPME was used as a more sensitive method for volatile analysis.

## 2. Materials and Methods

### 2.1. Plant Material

*Prunus mahaleb L.* seeds were obtained from Egypt, harvested in late summer during the year of 2019 [9]; in addition, commercial samples were obtained from Greece and Sudan, with sample information presented in Table 1. The powdered seeds were roasted by wrapping 5 g in aluminum foil and then heating them on a heating mantle at 180 °C for 15–20 min. A voucher specimen was deposited at the College of Pharmacy Herbarium, Cairo University, Cairo, Egypt. Each specimen was analyzed in triplicate by grinding separately 20 g of seeds to assess for biological variance.

### 2.2. SPME and Chemicals

SPME fibers of stable flex coated with divinylbenzene/carboxen/polydimethylsiloxane (DVB/CAR/PDMS, 50/30 µm) or PDMS (polydimethylsiloxane) were purchased from Supelco (Oakville, ON, Canada). All other chemicals, volatile standards and sugars were purchased from Sigma Aldrich (St. Louis, MO, USA).

### 2.3. GC–MS Analysis of Silylated Primary Metabolites

Primary metabolite analysis was carried out as follows. Briefly, 100 mg of finely powdered seeds (unroasted/roasted) was extracted with 5 mL 100% methanol with sonication for 30 min and frequent shaking, succeeded by centrifugation at 12,000× *g* for 10 min to remove debris. For evaluation of biological replicates, 3 different samples for each *P. mahaleb* seed accession were analyzed under the same conditions. Then, 100 µL of the methanol extract was aliquoted in screw-cap vials and left to evaporate under a nitrogen gas stream until complete dryness. For derivatization, 150 µL of *N*-methyl-*N*-(trimethylsilyl)-trifluoroacetamide (MSTFA) previously diluted 1/1% with anhydrous pyridine was mixed with the dried methanol extract and incubated for 45 min at 60 °C prior to analysis using GC–MS. Separation of silylated derivatives was achieved on a Rtx-5MS (30-m length, 0.25-mm inner diameter and 0.25-m film) [10]. The protocol to validate silylation was adopted as previously reported [11].

### 2.4. SPME–GC–MS Volatile Analysis

Dried, finely powdered seeds (unroasted/roasted) (20 mg) were placed in SPME screw-cap vials (1.5 mL) spiked with 10 µg (*Z*)-3-hexenyl acetate with fibers inserted manually above and placed in an oven kept at 50 °C for 30 min. HS-SPME analysis of the volatile compounds was performed as reported in [10] with slight modifications. The fiber was subsequently withdrawn into the needle and then injected manually into the injection port of a gas chromatography–mass spectrometer (GC–MS). GC–MS analysis was adopted on an Agilent 5977B GC/MSD equipped with a DB-5 column (30 m × 0.25 mm i.d. × 0.25 µm film thickness; Supelco) and coupled to a quadrupole mass spectrometer. The interface and the injector temperatures were both set at 220 °C. Volatile elution was carried out using the following gradient temperature program: oven was set at 40 °C for 3 min, then increased to 180 °C at a rate of 12 °C/min, kept at 180 °C for 5 min, finally increased at a rate of 40 °C/min to 240 °C and kept at this temperature for 5 min. Helium was utilized as a carrier gas with a total flow rate of 0.9 mL/min. For ensuring complete elution of volatiles, SPME fiber was prepared for the next analysis by placing it in the injection port at 220 °C for 2 min. For assessment of biological replicates, three different samples for each *P. mahaleb* seed accession were analyzed under the same conditions. Blank runs were made during sample analyses. The mass spectrometer was adjusted to EI mode at 70 eV with a scan range set at *m/z* 40–500.

### 2.5. Metabolite Identification and Multivariate Data Analyses

Identification of volatile and non-volatile silylated components was performed by comparing their retention indices (RI) in relation to n-alkanes (C6-C20), mass matching to NIST, WILEY library database and with standards if available. Peaks were first deconvoluted using AMDIS software (www.amdis.net (accessed on 28 November 2019)) [12] before mass spectral matching. Peak abundance data were exported for multivariate data analysis by extraction using MET-IDEA software (Broeckling, Reddy, Duran, Zhao, & Sumner, 2006). Data were then normalized to the amount of spiked internal standard (*Z*)-3-hexenyl acetate and then subjected to principal component analysis (PCA), hierarchical clustering analysis (HCA) and partial least squares discriminant analysis (OPLS-DA) using SIMCA-P version 13.0 software package (Umetrics, Umeå, Sweden). Markers were subsequently identified by analyzing the S-plot, which was declared with covariance (p) and correlation (pcor). All variables were mean-centered and scaled to Pareto variance. Model validation was assessed by computing the diagnostic indices, viz. Q2 and R2 values, and permutation testing.

## 3. Results and Discussion

The goal of this study was to explore the metabolome diversity within cultivars of white and red mahlab seeds obtained from Greece, Egypt and Sudan (Table 1) and to further determine roasting impact on their metabolite makeup, i.e., nutrient non-volatiles and aroma compounds, aided by chemometric tools. For the assessment of the biological variance within each specimen and analysis conditions, three independent biological replicates were simultaneously analyzed by GC–MS.

### 3.1. Primary Metabolite Profiling of Roasted/Unroasted Mahlab Seeds via GC–MS Analysis (Post-Silylation)

**Figure 1:**
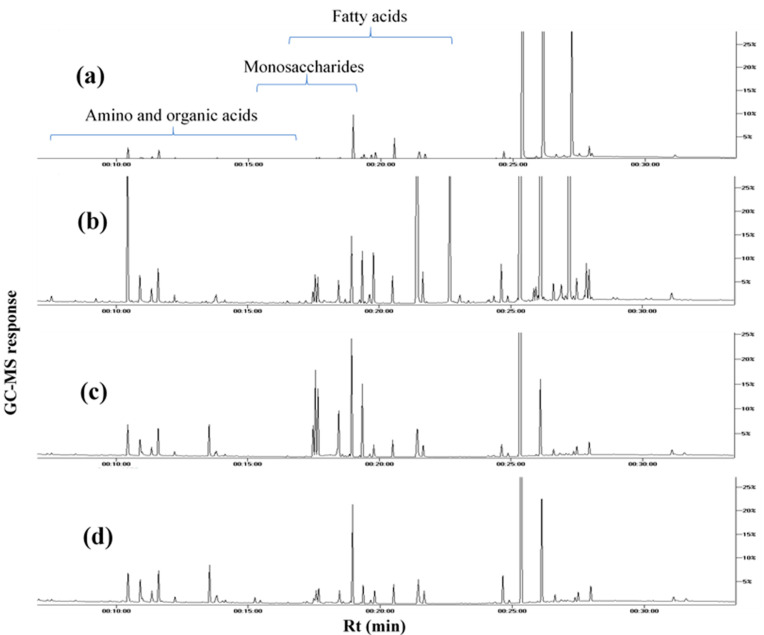
Representative GC–MS chromatograms of TMS derivatives of primary metabolites, collected from the extracts of (**a**) white mahlab from Greece “WG”, (**b**) white mahlab from Egypt “WE”, (**c**) red mahlab from Egypt “RE” and (**d**) roasted red mahlab from Egypt “RRE”.

To provide an overview of primary metabolites accounting for seeds’ nutritional value and organoleptic features (Table 1), GC–MS analysis was employed post-silylation. A total of 65 peaks (Table 2, Figure 1) were annotated, including sugars (mono- and di-saccharides), fatty acids, esters, organic acids and alcohols, in addition to a few amino acids and nitrogenous compounds.

#### 3.1.1. Sugars

Sugars, mostly represented by di-saccharides, were the most dominant primary metabolite class at levels ranging from 57.4 to 94.1%, comprising 30 peaks for 24 different sugars. Sucrose (peaks 51 and 52) was the main sugar detected in all samples, except for unroasted white mahlab from Egypt “WE” and Greece “WG”, as turanose (peaks 54 and 56) was the predominant sugar. White mahlab and red mahlab from Sudan, “WS” and “RS”, along with their roasted seeds, “RWS” and “RRS”, were characterized by an abundance of sucrose at 52.3–71.9%, compared to 15–23.7% in other samples. Turanose, a non-cariogenic isomer of sucrose, is present naturally in honey and exhibits a 50% sweetness level compared to that of sucrose [13]. Turanose abundance presents added value, being of low-glycemic response, and it has potential as a promising functional sweetener for controlling obesity [14]. Turanose was detected in all white mahlab seeds (11.4–32.6%) at much higher levels than in the red ones (0.1–0.6%), suggesting that it can function as a marker to distinguish between cultivars. Trehalose (peak 59) and cellobiose (peaks 63 and 64) content showed variation according to cultivar type or seed color. Monosaccharides in unroasted red mahlab from Egypt, “RE”, were the major sugars detected, at 29.7%, while the lowest level of monosaccharides was found in unroasted WS (0.8%). A significant decrease in monosaccharide levels was observed in both roasted RRE samples from Egypt and RRS from Sudan, dropping to 9.7% and 2.5%, respectively, likely due to the sugars’ utilization to generate Maillard products, which greatly influence seed color and flavor [15]. Major monosaccharides were fructose (peak 38), sorbose (peak 39) and glucose (peak 45) reaching 9.7, 7.8 and 7.5%, respectively, in RE.

Having higher thermal stability, sugar alcohols do not take part in any Maillard reaction and are regarded as low-glycemic-index sweeteners compared to free sugars [16]. Roasting increased sugar alcohols, with RRE appearing to be the most enriched, at 18.9%, followed by 14.4% for WG. Glucitol (peak 43) represented the major sugar alcohol in all specimens, except for WE, WS and RWS samples, which encompassed galactinol (peaks 55, 57, 60) as the most abundant sugar alcohol. Myo-Inositol (peaks 46 and 49), the most abundant inositol isomer in cereals, nuts, beans and fruits, improves insulin sensitivity [17]. Whether mahlab seed could represent a potential functional food for diabetic patients needs to be now investigated based on these results. Unroasted WE from Egypt has the best sugar profile owing to the presence of reduced levels of sucrose (15%).

#### 3.1.2. Organic Acids/Alcohols

Organic acids can enhance the digestive process and, alongside their salts, boost the utilization of protein in animals, besides their preservative action in food [18]. The higher levels of organic acids in unroasted RE and roasted red mahlab, “RRE”, from Egypt (5.9–9.7%), with an abundance of malic acid (4.7–7.4%), account for their sour taste compared to other samples. Succinic (peak 4), *γ*-hydroxybutyric (peak 3), oxalic (peak 2) and glycolic (peak 1) acids were also present in all mahlab samples. Malic acid (peak 5), a common acid in unripe fruit, is used to enhance beverages as a food acidulant [19]. *γ*-Hydroxybutyric acid, a natural neurotransmitter in the mammalian brain, is used therapeutically to treat alcoholism and narcolepsy [20]. 

It is noteworthy to mention that phytic acid, an anti-nutrient that interferes with the bioavailability of proteins and minerals owing to its strong chelating property, was absent from all seeds, indicating their good food quality [10]. 

Glycerol (0.3–7.4%) and glycerol-3-phosphate (peak 10) were identified as alcohols in all mahlab seeds except WG and RE. Although glycerol (peak 9) has a role in Maillard reactions as an active flavor precursor during roasting, its level interestingly showed an increase post-roasting, likely due to the increased lipid degradation [21].

#### 3.1.3. Amino Acids/Nitrogenous Compounds

Low amino acid levels at 0.05–0.60% in mahlab seeds were detected, represented by proline (peak 11), alanine (peak 12), pyroglutamic (peak 13) and glutamic (peak 14) acids. Mahlab seeds from Egypt, “WE”, “RE” and “RRE”, were the most enriched in β-ethanolamine (peak 31), an amino alcohol. 

#### 3.1.4. Fatty Acids/Acyl Esters/Sterols

Another considerable metabolite class present in all examined seeds was fatty acids, reaching 15.3% in white mahlab, “WE”, from Egypt, also accounting for its fatty taste. The monounsaturated omega-9 fatty acid, oleic acid (peak 23), was most enriched in WE (4.8%), making it the major fatty acid, indicating a heart-healthy functional food with diminished LDL (low-density lipoprotein) cholesterol [22]. An oleic-acid-rich diet has been reported to reduce the risk of atherosclerosis and type 2 diabetes [23]. The essential omega-3 polyunsaturated α-linolenic fatty acid (peak 25), detected at the highest levels in WE (4.1%), exhibits neuroprotective, anti-inflammatory, antioxidant and antidepressant effects [24]. White mahlab, “WE”, showed the highest omega 3/6 ratio among all seed accessions, providing the best fatty acid profile with the highest oleic acid content. The omega 3/6 ratio was observed to decline upon roasting, particularly in WS. α-Tocopherol (peak 65), a major constituent of vitamin E with a protective effect against atherosclerosis [25], and stigmast-5-ene were further detected in all mahlab seeds though at trace levels.

With regard to fatty acyl esters, the highest levels were detected in mahlab from Egypt, “WE”, “RE” and “RRE”, and Greece, “WG” (9–15.6%), with glycerol monostearate (peak 18) and n-butylcaprylate (peak 15) as major esters compared to 0.2–0.6% only in samples from Sudan. Glycerol monostearate is an anti-staling additive for bakery products that is widely used to improve the appearance and taste of flour foods and its abundance can account for the common use of mahlab seeds in bakery, aside from its role as flavor enhancer [26]. Generally, monoglycerides act as emulsifiers, yielding a more stable air-dispersed baked cake with a fairly soft crumb [27]. Another acyl ester of potential flavor described as fruity is n-butylcaprylate, detected at maximal levels in roasted red mahlab, “RRE” (6.5%), which is used in various foodstuffs, further rationalizing how roasting improves mahlab flavor [28]. 

#### 3.1.5. Inorganic Acids

Phosphoric acid (peak 27) was the only inorganic acid detected in higher levels in red mahlab seeds (1.8–3.6%) *versus* white types at 0.02–0.28%, suggesting that it can function as a marker to distinguish between mahlab cultivars of different colors. Phosphoric acid improves the antioxidant activity of α-tocopherol, aside from being a food preservative [29].

#### 3.1.6. Phenolics

The only identified phenolic compound was thymol-*β*-glucopyranoside (peak 33), which could serve as a precursor for thymol, the principal flavoring in mahlab. Many flavor and aroma components are bound to sugars in the form of glycosides. Glycosidically bound volatile compounds in plants are predominantly *O*-*β*-D-glucosides and *O*-diglycosides [30]. As most of the free forms consist of compounds with interesting flavor properties, their glycosides, acting as important potential aroma reserves, are usually greater than their free volatile counterparts. The odorous aglycones are released from precursors during various processing steps by enzymatic and non-enzymatic transformations [31].

### 3.2. HCA and PCA Analysis of Primary Metabolome of Unroasted White and Red Mahlab Seeds 

**Figure 2:**
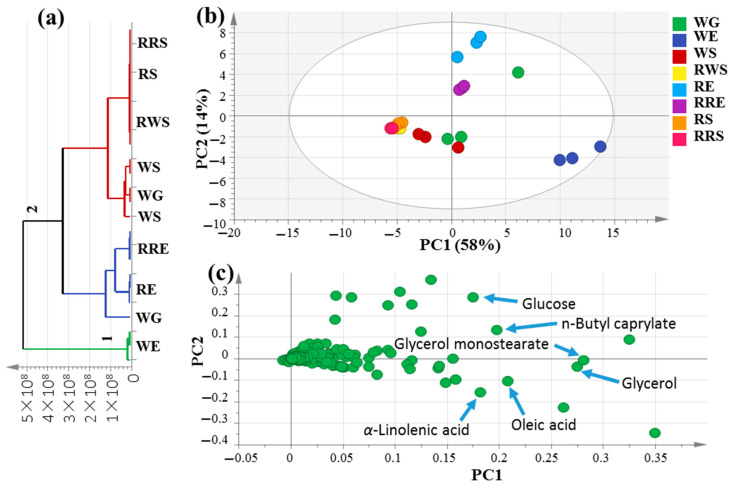
Unsupervised multivariate data analyses of the studied *P. mahaleb* seeds derived from modeling silylated primary metabolites analyzed via GC–MS (*n* = 3). (**a**) HCA plot. (**b**) PCA score plot of PC1 vs. PC2 scores. (**c**) The respective loading plot for PC1 and PC2, providing mass peaks and their assignments. The metabolome clusters are placed in two-dimensional space at the distinct locations defined by two vectors of principal component PC1 = 58% and PC2 = 14%.

Multivariate data analyses were conducted in order to better assess the primary metabolite heterogeneity among mahlab specimens in an unbiased manner using HCA and PCA. HCA acquired a dendrogram of three distinct clusters (Figure 2a), where white mahlab specimens from Egypt, “WE”, clustered in group 1, while other samples from Egypt, in addition to all seeds from Sudan and Greece, were separated into two subdivisions from group 2. However, the clustering of raw and roasted samples together in group 2 indicates that HCA failed to characterize the impact of roasting on mahlab seeds based on their silylated primary metabolite content. A PCA model (Figure 2b) was illustrated by two orthogonal PCs, justifying 72% of the entire variance, with the obvious discrimination of all mahlab seeds from Egypt at the right side of PC1, whereas RWS, RS and RRS were segregated in one intermixed cluster on the left side of PC1. Inspection of the loading plot in Figure 2c indicates that higher fatty acid levels were in agreement with the enriched content of oleic (peak 23) and *α*-linolenic (peak 25) acids in addition to glycerol (peak 9) in white mahlab seeds, also explaining its segregation on the lower right side of the loading plot. Moreover, WE seeds showed the highest content in lactones in the GC–MS analysis of volatiles, indicating a positive relationship between fatty acids and lactones. Roasted red mahlab seed “RRE” was characterized by high levels of *n*-butylcaprylate (peak 15) *versus* glucose (peak 25) abundance in unroasted RE seeds, likely be consumed in the Maillard reaction partially upon roasting. In contrast, glycerol monostearate (peak 18) was the dominant ester in white mahlab, “WG”, from Greece. 

### 3.3. OPLS-DA Analysis of Unroasted White and Red Mahlab Seeds

**Figure 3:**
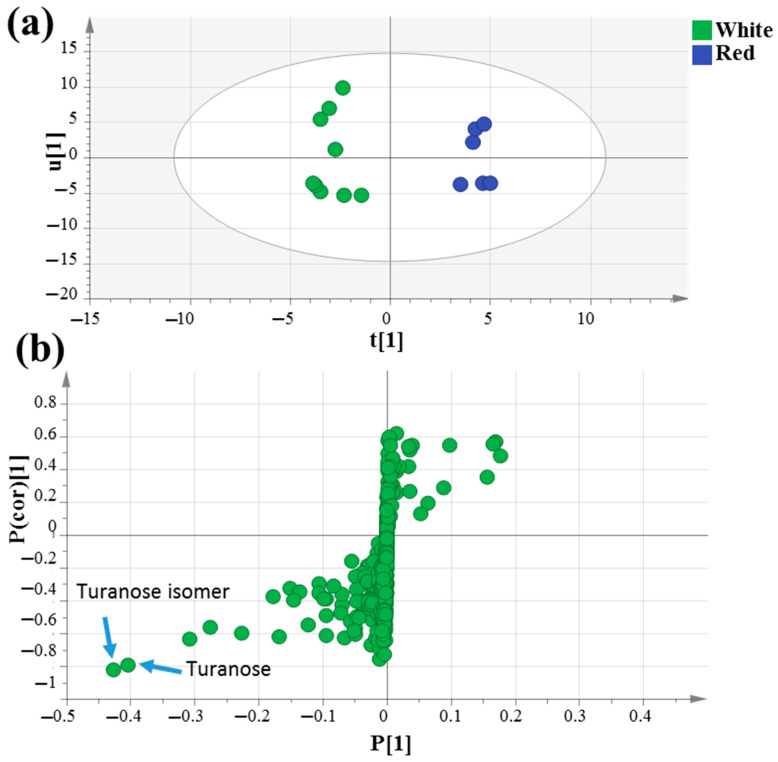
GC–MS-based OPLS-DA score plot (**a**) derived from modeling silylated primary metabolites of unroasted white *versus* red mahlab (*n* = 3). The respective loading S-plots (**b**) showing the covariance p [1] against the correlation p(cor) [1] of the variables of the discriminating component of the OPLS-DA model. Cut-off values of *p* < 0.005 were used. Designated variables are highlighted and identifications are discussed in the text.

The supervised orthogonal partial least squares discriminant analysis (OPLS-DA) score plot in Figure 3a was further employed to assess mahlab seed classification based on its cultivar type, which failed to separate in PCA analysis, by constructing a model of raw white “WG”, “WE” and “WS” against red “RE” and “RS” mahlab of different origins. OPLS-DA has a greater capability for marker recognition by supplying the most pertinent variables for the distinction of two class groups. With Q2 = 0.5 indicating the predictability of the model, the OPLS score plot demonstrated 59% of the total variance (R2 = 0.59). The respective loading S-plot (Figure 3b) revealed that turanose (peak 54) and its isomer (peak 56) were enriched in unroasted white mahlab compared to red ones, with *p* value < 0.005. 

### 3.4. Headspace Volatile Analysis Using SPME–GC–MS of Mahlab Samples (Roasted/Unroasted) 

Some mahlab seeds (roasted and unroasted), “WG”, “WE”, “RE” and “RRE”, were selected for volatile analysis based on their lower sugar content, as they are considered healthier foods, in addition to their higher ester levels, indicating a rich aroma. Headspace–solid-phase microextraction (HS-SPME) led to the identification of 135 volatiles belonging to several phytochemical classes, viz., organic acids, alcohols, aldehydes, esters, furans, ketones, lactones, aliphatic hydrocarbons, aromatics and sesqui- and monoterpenes (Table 3, Supplementary Figure S1).

#### 3.4.1. Aldehydes/Ketones

Aldehydes represented the major class of volatiles in specimens at ca. 37.34, 32.52, 44.60 and 19.48% in WG, WE, RE and RRE, respectively. Benzaldehyde (peak 36) was the most abundant aldehyde, being more enriched in RE samples (ca.18.71%), followed by (*E*)-cinnamaldehyde (peak 47), present in a relatively high amount at ca. 12.3, 11.2 and 7.2% in WE, RE and WG, respectively. The content of both aldehydes (peaks 36 and 47) was found at different levels among seed accessions, but most notably was found to decline upon roasting to reach 0.8 and 1.9%, respectively. Benzaldehyde is known to account for the characteristic smell of bitter almonds produced as a result of amygdaline glycoside hydrolysis [32] and it likely to lead to a similar scenario in mahlab seeds. (*E*)-Cinnamaldehyde exhibits a pungent cinnamon-like aroma and is likely to contribute to *P. mahaleb* seeds. The well-reported antimicrobial activity of (*E*)-cinnamaldehyde against a wide range of microorganisms [33] might contribute to the *P. mahaleb* seeds’ use as an antidiarrheal agent in folk medicine.

Unlike aldehydes, ketones were detected at much lower levels in all specimens, ca. 3.7, 2.5, 5.3 and 3.7% of the total volatiles in WG, WE, RE and RRE samples, respectively. Moreover, 3-Octen-2-one (peak 102) was the most abundant ketone, detected at the highest levels in WG and RE seeds (2.3 and 2.8%, respectively). 

#### 3.4.2. Lactones

Lactones constituted the second most abundant volatile class in mahlab seeds, with WE and WG yielding the highest levels (ca. 33.9 and 14.2%, respectively), and they increased upon roasting, being detected in RE at 6.8% to reach 24.2% in RRE seeds. Such an increase is likely due to the dehydration impact of roasting to enhance the intramolecular lactonization of some compounds hydroxy fatty acids producing *γ* and *δ*-lactone compounds [34]. WE also encompassed, e.g., the highest fatty acid level, indicating that lactones originated from fatty acid cyclization. Among the identified lactones, *γ*-caprolactone (peak 114), isomintlactone (peak 118) and *γ*-nonalactone (peak 119) showed increases from 2.14, 1.19 and 0.88% to 6.58, 8.31 and 4.20%, respectively, upon roasting in RE samples. Such observations can explain why the traditional use of mahlab seeds as flavoring agents in confections implies a short heating process to improve its sensory attributes, as evidenced by the increased lactone-derived aroma. *γ*-Caprolactone has sweet herbaceous notes [35], while isomintlactone and *γ*-nonalactone are characterized by mint-like [36] and coconut flavors [37], respectively.

Coumarin (peak 122) along with herniarin (7-methoxycoumarin) (peak 123) were found to be enriched in WE samples, at 19.75 and 1.37%, respectively, compared to the other samples. Coumarin is well-known for its pleasant vanilla-like odor and slightly bitter taste and was previously reported in most parts of the *P. mahaleb* tree, i.e., leaves, fruits, seeds and wood, as well as in other plants, e.g., *Melilotus alba* and *Cinnamonum verum* [38]. Aside from their strong aroma, coumarins are reported to exhibit a wide range of biological actions, viz. antioxidant, anticancer, anti-inflammatory and antimicrobial actions [39]. However, hepatic damage and carcinogenic effects were also reported in experimental animals, limiting their potential use in humans. Accordingly, a tolerable daily intake of 0.1 mg/kg of body weight has been estimated based on human data experiments and recommended by the Scientific Panel on Food Additives, Flavorings, Processing Aids and Materials in Contact with Food (AFC) to guard against any hepatotoxicity [40]. Absolute quantification of coumarins in *P. mahaleb* should now follow, using standards in order ensure conclusive results.

#### 3.4.3. Acids/Esters

The relative amounts of both organic and fatty acids detected in the aroma of white mahlab seeds obtained from Greece and Egypt and red Egyptian mahlab unroasted seeds were ca. 22.47, 19.12 and 17.43%, respectively. Greek mahlab seeds, “WG”, were the most abundant in acids, with valeric acid and its isomer (peaks 5 and 6) accounting for 9.2% among other acids. Valeric acid is a low-molecular-weight carboxylic acid with a very unpleasant odor, which commonly occurs in the valerian flowering plant [41]. It was previously reported in another *Prunus* species, namely Japanese apricot (*P. mume*) [42]; however, this is the first report of its detection in *P. mahaleb.* This acid is primarily used in the synthesis of esters to generate pleasant odors (fruity flavors) used in perfumes, cosmetics and food additives such as ethyl valerate and pentyl valerate [43]. The latter ester and its respective isomer (peaks 76 and 80) were detected at comparable levels as the most abundant esters at 1.6–1.9% in WG samples, respectively, and 1.2–1.6% in red Egyptian mahlab samples “RE”, respectively, and are likely derived from valeric acid, also present in seeds. Other esters present at lower levels included pentyl caproate and n-butyl acetate (peaks 82 and 71) at 0.95 and 0.87%, respectively, in WG samples. An increase in the pentyl caproate level in RE was observed upon roasting from 0.41 to 2.25%, which would likely intensify its flavor. 

Several short-chain fatty acids were identified, including butanoic acid, for the first time in *P. mahaleb*, reported before in apricot (*P. armeniaca*), in addition to caprylic and caproic acid, which were previously only reported in *P. mume* [42]. 

#### 3.4.4. Alcohols

In contrast to aldehydes showing differences among specimens, alcohols were detected at comparable levels of 3–6% in WG, WE, RE and RRE samples. In particular, 3-Octenol (peak 27), 1-hexanol (peak 25) and 2,3-butanediol (peak 23) represented the major forms in RE at ca. 1.62, 1.45, and 0.98%, respectively, and decreased upon roasting (0.97, 0.07 and 0.11%, respectively); the exception was 1-octanol (peak 28), which showed a predominance in roasted sample RRE at 3.2%.

Moreover, 1-Hexanol (peak 25) was reported to impart green, sweet notes in lychee fruits and red wines, whereas pentanol (peak 22) was reported to contribute to the characteristic aroma of mahlab seeds [44].

#### 3.4.5. Furans

Very low amounts of furan and its derivatives were detected, found at the highest levels in WG (5.41%), followed by 2.4 and 3.8% in WE and RE samples, respectively. As expected, furan increased up to 6.37% upon roasting of RE samples, likely resulting from sugar oxidation in the seeds [45]. Furthermore, 2-Butylfuran (peak 95) and 2-pentylfuran (peak 96) were the most abundant furans in WG at 1.5 and 2.6%, respectively. Furan and its derivatives, i.e., 2-alkylfurans, 2-acetylfuran, furfural and furfuryl alcohol, are produced via Maillard reaction in many heat-processed foods and beverages to contribute to their sensory properties. Furans can also serve as indicators of time–temperature effects during the production and storage of food items. In addition, 2-Akylfurans such as 2-butyl (peak 95) and 2-pentylfurans (peak 96) are mainly formed from lipid degradation [46], and some impart a mild, sweet and fruity taste, including 2-butylfuran, a constituent of many plant species and some cooked foods [47].

#### 3.4.6. Ethers

Ethers were detected at trace levels (0.01–0.05%), represented mostly by estragole (peak 90), a common flavor in many herbs and spices. Estragole, closely related to safrole, is a hepatotoxic and hepatocarcinogenic compound in experimental animals [48]; however, it is unlikely to exert any deleterious effects in *P. mahaleb*, being detected at such trace levels. 

#### 3.4.7. Mono- and Sesquiterpene Hydrocarbons

Mono- and sesquiterpenes amounted for 4.7 and 0.4% of the total volatiles in RE samples, found most enriched in the former compounds, suggesting that, in general, monoterpene biosynthesis is more strongly activated than that of sesquiterpenes in *P. mahaleb*. Mono- and sesquiterpenes were detected at 0.3–0.6% and 0.1–0.3% in other seeds, respectively. Limonene (peak 124) was the major monoterpene in RE, at 4.3%, and it declined upon roasting to reach 0.1%. Limonene is a key flavor in citrus fruits, with a pleasant lemon-like odor, aside from its several biological effects [49]. Its abundance in RE means that this specimen has the most favored aroma and it has yet to be evaluated using sensory analysis.

#### 3.4.8. Aliphatic Hydrocarbons

Aliphatic hydrocarbons accounted for only 0.5–0.9% of the volatile composition in all seeds. Dodecane (peak 58), pentadecane (peak 59), tetradecane (peak 62) and hexadecane (peak 63) were prevalent, whereas heptadecane (peak 64) and nonadecane (peak 66) were detected at trace levels, previously identified in mahlab flower volatiles to serve as pheromones [50].

#### 3.4.9. Aromatics 

Among aromatics, naphthalene (peak 68), with its strong tar-like odor [51], was detected exclusively in roasted seeds, though at a low level of 2.7%. Naphthalene and other PAHs (Polycyclic aromatic hydrocarbons) were reported to be formed during thermal food processing such as roasting, frying, grilling, toasting and smoking [52] and can thus be used as a marker for heat effects in *P. mahaleb* according to these results. 

### 3.5. PCA Analysis of the Aroma Profiles of Unroasted White and Red Mahlab Seeds from Greece and Egypt

**Figure 4:**
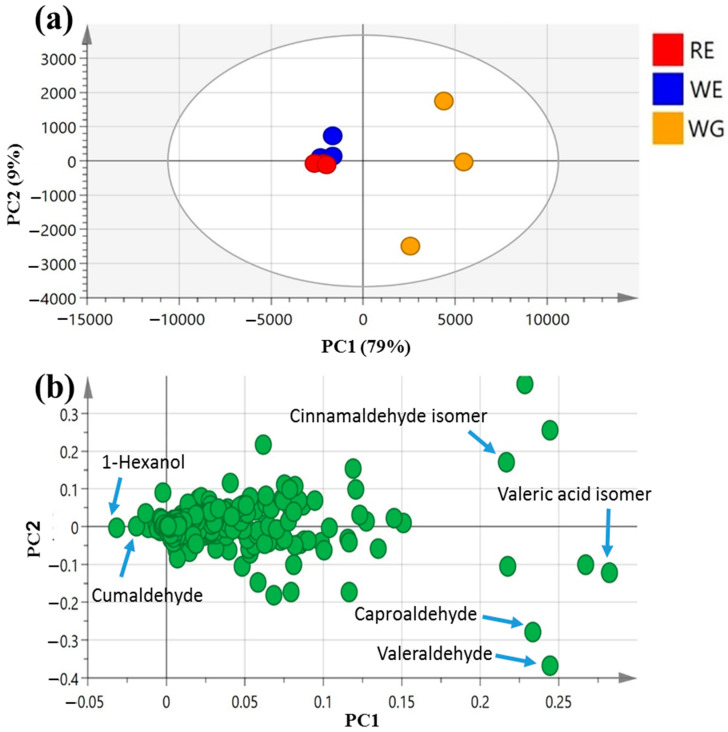
Unsupervised multivariate data analyses of the studied mahlab seeds derived from modeling volatile profiles analyzed via GC–MS (*n* = 3). (**a**) PCA score plot of PC1 vs. PC2 scores. (**b**) The respective loading plot for PC1 and PC2, providing their assignments. The metabolome clusters are placed in two-dimensional space at the distinct locations defined by two vectors of principal component PC1 = 79% and PC2 = 9%.

Heterogeneity in the volatile distribution of seed accessions was explored in a more holistic way using PCA, as in the primary plant metabolite dataset (Figure 4) [53]. 

PCA scores of the mahlab seed aroma profile (Figure 4a) were ascribed by two main vectors, i.e., PC1 and PC2, accounting for 88% of the total variance among samples. White WE and red RE mahlab seeds obtained from Egypt were clustered together along the negative side of PC1, apart from white Greek “WG”, suggesting that geographical origin overcomes cultivar type with regard to the analyzed seeds’ aromas.

The main volatiles responsible for specimens’ segregation were revealed by the PCA loading plot (Figure 4b) to be 1-hexanol (peak 25) and cumaldehyde (peak 52), being most enriched in both WE and RE. In contrast, valeraldehyde (peak 29), with a pungent, bitter, almond-like odor [54] (peak 29); caproaldehyde, a with tallowy, green, leafy aroma [54] (peak 31) and isomeric forms of cinnamaldehyde (peak 47) and valeric acid (peak 6) predominated among volatiles in mahlab seeds from Greece, suggesting that Greek specimens exhibited better aromas, being abundant in aldehydes. 

### 3.6. OPLS-DA Analysis of Unroasted versus Roasted Red Mahlab Seeds

OPLS-DA modeling of the volatile profile of unroasted RE *versus* roasted red mahlab “RRE” seeds (Supplementary Figure S2) was attempted in order to assess the impact of roasting on their aroma as well as to identify roasting markers from the volatile dataset. The performance of the developed classification model was validated by the computed parameters “R2 (0.9621)” and “Q2 (0.9102)”, which indicated the optimal fit and prediction power of the model, respectively, though with a *p* value > 0.05, suggestive of non-statistical significance. 

The observed segregation in the derived score plot (Supplementary Figure S2a) was ascribed to the enrichment of unroasted RE in 1-hexanol (peak 25) and benzaldehyde (peak 36) *versus* the abundance of lactones, e.g., *γ*-caprolactone (peak 114) and isomintlactone (peak 118); furans, e.g., 1-(2-furyl)-3-butene-1,2-diol (peak 99), and a polyaromatic, i.e., naphthalene (peak 68) in roasted mahlab seeds, likely produced due to the applied high temperature during roasting (as depicted in S-plot, Supplementary Figure S2b). The production of lactones, furans and polyaromatic compounds is expected at high temperatures [55] and could indeed reveal the roasting impact on mahlab seed aroma.

## 4. Conclusions

The compositional heterogeneity in the primary and aroma metabolite profiles of *P. mahlab* seeds in the context of cultivar type and roasting is presented herein for the first time through a holistic untargeted GC–MS metabolomics coupled with multivariate data analyses.

The results of GC–MS analysis after silylation led to the detection of 65 peaks, including mostly sugars, fatty acids and acyl esters, with considerable amounts of glycerol, which increased post-roasting. Turanose was detected as the most abundant sugar in white cultivars from Greece and Egypt, while sucrose was predominant in all Sudanese specimens. Multivariate data analysis of the primary metabolome revealed that white mahlab from Egypt was the most distinct among accessions with the highest fatty acid level, viz., oleic and α-linolenic acids, contributing to its fatty taste. 

Around 135 volatiles were identified in mahlab seeds (roasted and unroasted) belonging to organic acids, aldehydes, alcohols, esters, furans, ketones, lactones, aliphatic hydrocarbons, aromatics and terpenes, with the first two classes most abundant in all seed accessions. White mahlab from Egypt showed the best aroma, being abundant in cinnamaldehyde (peak 47), concurrent with the highest lactone level, with a sweet taste that could compensate for its relatively low sugar content.

The selected mahlab resources in this study were collected from three countries that traditionally use mahlab and other worldwide varieties are yet to be included. Our approach is certainly applicable to the analysis of samples from such further sources for exploiting factors that might influence seed metabolic profile, i.e., seasonal variation, growth stage, storage and other processing conditions.

The most discriminating volatile class was aldehydes to distinguish seeds of different origin. With regard to volatile markers in the roasting process, 1-octanol, *γ*-caprolactone and isomintlactone, as well as furans, e.g., 1-(2-furyl)-3-butene-1,2-diol and naphthalene, were enriched in the roasted seeds. A positive correlation was observed in all mahlab seed accessions among lactone level and fatty acids, likely via cyclization. Considering the lipid profile, white mahlab from Egypt presented the highest omega-3 to -6 ratio, with additional high levels of omega-9 oleic acid. Concurrent with its lower sugar composition, it has promise as a healthier food, especially for obese people.

An extended approach utilizing liquid chromatography coupled to mass spectrometry (LC-MS) can be applied to pinpoint differences in bioactive secondary metabolite profiles among mahlab seed accessions and to further elucidate the health effects of *P. mahaleb*. Our study provided the first complementary phytochemical evidence that supports the nutritional and flavor determinants of mahlab seeds, although it has yet to be complemented by other functional components of mahlab seeds to corroborate their utilization as functional foods or further as nutraceuticals.

## Figures and Tables

**Table 1:** Origins of the different *P. mahaleb* seed accessions used in analysis.

Sample Codes	Seed Color	Location
WG	White	Greece (Athens)
WE	White	Egypt (Cairo)
WS	White	Sudan (Khartoum)
RWS	Roasted white	Sudan (Khartoum)
RE	Red	Egypt (Cairo)
RRE	Roasted red	Egypt (Cairo)
RS	Red	Sudan (Khartoum)
RRS	Roasted red	Sudan (Khartoum)

**Table 2:** Relative percentage of silylated metabolites in *P. mahaleb* seeds analyzed via GC–MS, *n* = 3. For codes, refer to Table 1.

No.	Rt (min)	KI	Name	WG	WE	WS	RWS	RE	RRE	RS	RRS
**Organic acids**											
1	7.062	1084.3	Glycolic acid (2TMS)	0.02 ± 0.01	0.04 ± 0.01	0.01 ± 0.01	0.33 ± 0.28	0.05 ± 0.01	0.33 ± 0.02	0.02 ± 0.01	0.04 ± 0.01
2	7.531	1111.8	Oxalic acid (2TMS)	0.16 ± 0.08	0.55 ± 0.01	0.09 ± 0.07	0.18 ± 0.09	0.30 ± 0.04	0.59 ± 0.21	0.15 ± 0.01	0.25 ± 0.11
3	9.764	1241.9	*γ*-Hydroxybutyric acid (2TMS)	0.04 ± 0.02	0.17 ± 0.01	0.01 ± 0.01	0.04 ± 0.01	0.11 ± 0.01	0.23 ± 0.03	0.03 ± 0.01	0.07 ± 0.03
4	10.980	1321.4	Succinic acid (2TMS)	0.04 ± 0.01	0.10 ± 0.01	0.02 ± 0.01	0.08 ± 0.03	0.36 ± 0.02	0.57 ± 0.06	0.24 ± 0.06	0.37 ± 0.23
5	13.526	1500.9	Malic acid (3TMS)	0.23 ± 0.27	0.16 ± 0.07	0.02 ± 0.01	0.04 ± 0.02	4.73 ± 0.18	7.43 ± 0.35	0.32 ± 0.11	0.48 ± 0.13
6	14.127	1546.5	Unknown acid	0.06 ± 0.02	0.13 ± 0.01	-	0.01 ± 0.00	0.12 ± 0.01	0.20 ± 0.01	0.01 ± 0.00	0.02 ± 0.01
7	17.210	1800.1	Terephthalic acid (2TMS)	0.04 ± 0.01	0.15 ± 0.05	0.02 ± 0.01	0.15 ± 0.02	0.08 ± 0.01	0.28 ± 0.05	0.12 ± 0.02	0.15 ± 0.03
8	18.699	1941.2	Unknown acid	0.32 ± 0.42	0.27 ± 0.13	0.03 ± 0.02	0.12 ± 0.03	0.13 ± 0.03	0.05 ± 0.01	1.95 ± 0.26	0.13 ± 0.01
Total organic acids				0.90	1.58	0.20	0.96	5.88	9.69	2.84	1.51
**Alcohols**											
9	10.429	1285.9	Glycerol (3TMS)	2.96 ± 0.48	7.41 ± 0.66	0.32 ± 0.17	0.53 ± 0.14	3.75 ± 0.22	5.01 ± 0.39	5.36 ± 1.26	6.88 ± 3.69
10	16.966	1780.1	Glycerol-3-phosphate (4TMS)	-	0.05 ± 0.01	0.01 ± 0.01	0.03 ± 0.00	-	0.01 ± 0.00	0.01 ± 0.00	0.02 ± 0.00
Total alcohols				2.96	7.46	0.34	0.56	3.75	5.02	5.37	6.89
**Amino acids**											
11	10.763	1307.5	Proline (2TMS)	0.02 ± 0.00	0.13 ± 0.01	0.04 ± 0.03	0.01 ± 0.00	-	-	0.19 ± 0.06	0.07 ± 0.01
12	12.649	1434.9	Alanine (3TMS)	0.02 ± 0.01	0.04 ± 0.00	-	-	0.04 ± 0.00	0.06 ± 0.01	-	0.01 ± 0.00
13	14.009	1537.2	Pyroglutamic acid (2TMS)	0.06 ± 0.03	0.17 ± 0.01	0.01 ± 0.01	0.06 ± 0.01	0.15 ± 0.02	0.46 ± 0.06	0.34 ± 0.09	0.51 ± 0.05
14	15.183	1629.1	Glutamic acid (3TMS)	0.02 ± 0.01	0.06 ± 0.00	-	-	0.01 ± 0.00	-	0.01 ± 0.00	0.01 ± 0.00
Total amino acids				0.11	0.40	0.05	0.07	0.20	0.53	0.54	0.60
**Esters**											
15	11.590	1361.3	*n*-Butylcaprylate	2.90 ± 0.61	3.61 ± 0.22	0.01 ± 0.00	0.02 ± 0.00	4.43 ± 0.58	6.47 ± 0.34	0.01 ± 0.00	0.03 ± 0.01
16	24.625	2600.2	1-Monopalmitin (2TMS)	0.71 ± 0.04	2.08 ± 0.27	0.08 ± 0.04	0.06 ± 0.01	0.72 ± 0.05	1.76 ± 0.31	0.05 ± 0.01	0.09 ± 0.01
17	25.942	2767	1-Monooleoylglycerol (2TMS)	0.03 ± 0.00	0.42 ± 0.02	0.13 ± 0.05	0.17 ± 0.01	0.06 ± 0.01	0.11 ± 0.01	0.14 ± 0.02	0.29 ± 0.03
18	26.096	2777	Glycerol monostearate (2TMS)	11.73 ± 2.67	7.20 ± 0.76	0.37 ± 0.28	0.08 ± 0.01	3.76 ± 0.86	7.29 ± 1.22	0.02 ± 0.01	0.05 ± 0.01
Total esters				15.37	13.30	0.58	0.33	8.96	15.62	0.22	0.45
**Fatty acids**											
19	17.731	1848.8	Myristic acid (TMS)	0.02 ± 0.00	0.07 ± 0.01	0.01 ± 0.01	0.02 ± 0.00	0.04 ± 0.00	0.07 ± 0.01	0.02 ± 0.01	0.03 ± 0.01
20	19.773	2045.7	Palmitic acid (TMS)	0.63 ± 0.09	2.03 ± 0.18	1.02 ± 0.84	0.49 ± 0.09	0.80 ± 0.04	1.19 ± 0.11	0.88 ± 0.18	1.36 ± 0.12
21	20.737	2144.6	Margaric acid (TMS)	0.01 ± 0.00	0.04 ± 0.01	0.01 ± 0.00	0.02 ± 0.00	0.01 ± 0.00	0.02 ± 0.00	0.01 ± 0.00	0.03 ± 0.01
22	21.405	2215.3	Linoleic acid (TMS)	0.21 ± 0.00	2.87 ± 0.18	0.38 ± 0.19	0.34 ± 0.03	0.55 ± 0.06	0.60 ± 0.06	1.13 ± 0.20	1.86 ± 0.16
23	21.432	2219.3	Oleic acid (TMS)	0.62 ± 0.02	4.78 ± 0.90	1.73 ± 1.22	1.27 ± 0.06	1.34 ± 0.01	1.80 ± 0.21	2.29 ± 0.17	4.18 ± 0.53
24	21.649	2242.8	Stearic acid (TMS)	0.54 ± 0.04	1.28 ± 0.12	0.69 ± 0.52	0.34 ± 0.07	0.77 ± 0.04	1.17 ± 0.11	0.24 ± 0.03	0.55 ± 0.13
25	22.670	2357	*α*-Linolenic acid (TMS)	0.04 ± 0.00	4.11 ± 0.10	0.10 ± 0.02	0.05 ± 0.02	0.02 ± 0.00	0.02 ± 0.00	0.09 ± 0.01	0.14 ± 0.01
26	23.378	2441.3	Arachidic acid (TMS)	0.01 ± 0.00	0.08 ± 0.01	0.07 ± 0.05	0.04 ± 0.01	0.02 ± 0.00	0.03 ± 0.01	0.04 ± 0.01	0.08 ± 0.02
Total fatty acids				2.08	15.26	4.01	2.55	3.55	4.90	4.70	8.22
**Inorganic acids**											
27	10.454	1286.7	Phosphoric acid (3TMS)	0.28 ± 0.33	0.28 ± 0.08	0.02 ± 0.01	0.16 ± 0.01	1.80 ± 0.16	3.35 ± 0.40	2.94 ± 0.63	3.59 ± 1.67
Total inorganic acids				0.28	0.28	0.02	0.16	1.80	3.35	2.94	3.59
**Nitrogenous compounds**											
28	8.458	1163	Unknown nitrogenous compound	0.09 ± 0.04	0.21 ± 0.01	-	0.01 ± 0.00	0.20 ± 0.02	0.31 ± 0.02	0.02 ± 0.00	0.02 ± 0.01
29	10.899	1316.1	Unknown nitrogenous compound	0.33 ± 0.14	0.86 ± 0.04	0.18 ± 0.08	0.34 ± 0.09	0.79 ± 0.06	1.39 ± 0.07	0.19 ± 0.05	0.27 ± 0.15
30	11.269	1340.3	Pipecolic acid (2TMS)	0.03 ± 0.01	0.20 ± 0.01	0.05 ± 0.04	0.01 ± 0.00	-	-	0.01 ± 0.00	0.01 ± 0.00
31	12.214	1402.1	Ethanolamine (3TMS)	0.35 ± 0.10	0.86 ± 0.05	-	-	0.68 ± 0.07	1.04 ± 0.05	-	-
32	14.997	1613.2	Tyramine (TMS)	0.01 ± 0.00	0.03 ± 0.00	0.01 ± 0.00	0.02 ± 0.00	0.02 ± 0.00	0.04 ± 0.01	0.07 ± 0.02	0.09 ± 0.01
Total nitrogenous compound				0.81	2.15	0.23	0.37	1.69	2.78	0.29	0.40
**Phenolics**											
33	26.862	2889	Thymol-*β*-glucopyranoside (4TMS)	0.17 ± 0.08	0.40 ± 0.01	0.48 ± 0.06	0.59 ± 0.07	0.16 ± 0.04	0.25 ± 0.02	0.24 ± 0.01	0.56 ± 0.19
Total phenolics				0.17	0.40	0.48	0.59	0.16	0.25	0.24	0.56
**Sterols**											
34	31.098	3420.2	Stigmast-5-ene (TMS)	0.26 ± 0.01	0.28 ± 0.01	0.43 ± 0.12	0.33 ± 0.04	0.31 ± 0.04	0.43 ± 0.03	0.24 ± 0.04	0.53 ± 0.06
Total sterols				0.26	0.28	0.43	0.33	0.31	0.43	0.24	0.53
**Sugars**											
35	15.242	1634	Arabinose (4TMS)	-	-	0.01 ± 0.00	0.22 ± 0.03	0.01 ± 0.00	0.36 ± 0.04	0.02 ± 0.01	0.08 ± 0.00
36	15.433	1651.1	Ribono-1,4-lactone (3TMS)	0.02 ± 0.01	0.02 ± 0.00	0.01 ± 0.01	0.11 ± 0.02	0.02 ± 0.00	0.59 ± 0.05	0.03 ± 0.01	0.15 ± 0.01
37	16.497	1740.5	Arabitol (5TMS)	0.02 ± 0.01	0.08 ± 0.01	0.01 ± 0.00	0.03 ± 0.00	0.05 ± 0.00	0.09 ± 0.01	0.11 ± 0.03	0.11 ± 0.00
38	17.559	1833.7	Fructose (5TMS)	2.87 ± 3.51	1.66 ± 0.68	0.10 ± 0.05	0.36 ± 0.03	9.74 ± 0.29	2.30 ± 0.16	6.04 ± 1.21	0.49 ± 0.04
39	17.657	1843	Sorbose (5TMS)	1.08 ± 0.89	1.27 ± 0.55	0.06 ± 0.03	0.21 ± 0.01	7.78 ± 0.50	2.34 ± 0.08	3.44 ± 0.40	0.32 ± 0.03
40	18.376	1909.4	Galactonic acid, *γ*-lactone (4TMS)	0.95 ± 0.41	0.35 ± 0.13	0.03 ± 0.01	0.10 ± 0.01	5.84 ± 0.11	1.10 ± 0.68	0.44 ± 0.16	0.58 ± 0.04
41	18.445	1917.1	Mannopyranose (5TMS)	2.00 ± 2.39	1.10 ± 0.43	0.07 ± 0.03	0.46 ± 0.03	4.65 ± 0.13	1.77 ± 0.11	1.05 ± 0.20	0.15 ± 0.02
42	18.857	1956	Mannitol (6TMS)	0.05 ± 0.03	0.11 ± 0.01	0.02 ± 0.01	0.05 ± 0.00	0.28 ± 0.02	0.38 ± 0.02	1.11 ± 0.27	1.22 ± 0.07
43	18.938	1963.6	Glucitol (6TMS)	7.66 ± 0.71	3.27 ± 0.80	0.10 ± 0.05	0.15 ± 0.05	9.98 ± 0.49	11.62 ± 0.85	4.39 ± 0.73	4.84 ± 0.21
44	19.248	1993	Galactofuranose (5TMS)	0.97 ± 0.91	0.21 ± 0.05	0.09 ± 0.03	0.28 ± 0.01	0.11 ± 0.02	0.17 ± 0.02	0.96 ± 0.16	1.13 ± 0.06
45	19.343	2002	Glucose (5TMS)	3.45 ± 3.44	2.55 ± 0.71	0.46 ± 0.12	1.09 ± 0.11	7.45 ± 0.14	2.79 ± 0.18	1.54 ± 0.29	0.37 ± 0.03
46	19.449	2012.9	Myo-Inositol (6TMS)	0.52 ± 0.42	0.08 ± 0.02	0.10 ± 0.03	0.23 ± 0.02	0.05 ± 0.01	0.07 ± 0.01	0.62 ± 0.12	0.82 ± 0.05
47	19.582	2026.2	Ribonic acid (5TMS)	0.08 ± 0.04	0.06 ± 0.02	0.03 ± 0.01	0.04 ± 0.00	0.04 ± 0.01	0.03 ± 0.01	0.10 ± 0.02	0.12 ± 0.01
48	19.625	2031.1	Gluconic acid (6TMS)	1.22 ± 0.25	0.55 ± 0.11	0.04 ± 0.02	0.08 ± 0.03	0.38 ± 0.02	0.58 ± 0.06	0.72 ± 0.10	0.81 ± 0.06
49	20.497	2120.9	Myo-Inositol (6TMS) isomer	5.06 ± 2.13	1.36 ± 0.24	0.11 ± 0.03	0.28 ± 0.02	1.71 ± 0.03	2.59 ± 0.16	0.98 ± 0.33	1.56 ± 0.19
50	24.112	2535.5	Unknown sugar	0.02 ± 0.01	0.06 ± 0.01	0.04 ± 0.00	0.07 ± 0.01	0.04 ± 0.01	0.08 ± 0.01	0.01 ± 0.00	0.04 ± 0.01
51	24.344	2564.5	Sucrose (8TMS)	0.13 ± 0.02	0.35 ± 0.01	0.89 ± 0.15	0.72 ± 0.39	0.12 ± 0.02	0.24 ± 0.08	0.25 ± 0.08	0.79 ± 0.38
52	25.368	2687.2	Sucrose (8TMS) isomer	21.12 ± 8.53	14.65 ± 1.33	51.40 ± 5.08	71.13 ± 2.20	21.54 ± 0.54	23.44 ± 1.62	57.87 ± 3.23	58.43 ± 7.32
53	25.863	2757	Unknown Sugar	0.35 ± 0.01	0.78 ± 0.04	0.61 ± 0.10	0.57 ± 0.07	0.01 ± 0.00	0.03 ± 0.01	0.06 ± 0.05	0.15 ± 0.11
54	26.179	2788.8	Turanose (8TMS)	9.01 ± 0.98	7.60 ± 0.54	8.91 ± 7.69	1.66 ± 0.06	0.27 ± 0.03	0.45 ± 0.04	0.05 ± 0.01	0.11 ± 0.03
55	26.606	2851.2	Galactinol (9TMS)	0.39 ± 0.15	1.28 ± 0.04	1.85 ± 0.34	1.99 ± 0.09	0.66 ± 0.12	1.28 ± 0.14	0.66 ± 0.13	1.19 ± 0.13
56	27.189	2926.6	Turanose, (8TMS) isomer	16.76 ± 3.62	14.44 ± 1.13	23.63 ± 3.94	9.75 ± 0.30	0.17 ± 0.02	0.14 ± 0.02	0.08 ± 0.01	0.19 ± 0.07
57	27.366	2947.5	Galactinol (9TMS) isomer 1	0.15 ± 0.03	0.24 ± 0.02	0.15 ± 0.04	0.15 ± 0.04	0.35 ± 0.03	0.45 ± 0.01	0.07 ± 0.00	0.22 ± 0.07
58	27.486	2962.8	Melibiose (8TMS)	0.52 ± 0.21	1.52 ± 0.09	3.30 ± 1.14	2.38 ± 0.06	0.73 ± 0.17	1.34 ± 0.16	0.90 ± 0.23	1.29 ± 0.18
59	27.856	3009.6	Trehalose (7TMS)	1.84 ± 0.17	2.33 ± 0.14	0.63 ± 0.18	1.12 ± 0.14	0.03 ± 0.00	0.04 ± 0.00	0.05 ± 0.01	0.13 ± 0.01
60	27.960	3022.7	Galactinol (9TMS) isomer 2	0.62 ± 0.15	2.32 ± 0.07	0.52 ± 0.12	0.57 ± 0.04	1.26 ± 0.24	2.44 ± 0.27	0.20 ± 0.02	0.49 ± 0.10
61	28.885	3139.7	Unknown Sugar	0.03 ± 0.00	0.15 ± 0.01	0.08 ± 0.02	0.10 ± 0.02	0.04 ± 0.01	0.08 ± 0.01	0.04 ± 0.01	0.07 ± 0.01
62	30.321	3321.9	Unknown Sugar	0.03 ± 0.00	0.12 ± 0.01	0.06 ± 0.01	0.08 ± 0.00	0.04 ± 0.00	0.08 ± 0.01	0.08 ± 0.02	0.15 ± 0.02
63	31.202	3433	Cellobiose (8TMS)	0.02 ± 0.00	0.07 ± 0.01	0.05 ± 0.01	0.06 ± 0.01	0.09 ± 0.03	0.19 ± 0.03	0.47 ± 0.10	0.78 ± 0.11
64	31.567	3479.5	Cellobiose (8TMS) isomer	0.01 ± 0.00	0.03 ± 0.00	0.02 ± 0.00	0.03 ± 0.01	0.22 ± 0.02	0.39 ± 0.03	0.17 ± 0.03	0.28 ± 0.03
Total sugars				76.96	58.63	93.38	94.05	73.65	57.44	82.51	77.05
**TocSopherols**											
65	27.787	3001.1	*α*-Tocopherol (TMS)	0.12 ± 0.01	0.26 ± 0.02	0.28 ± 0.03	0.03 ± 0.00	0.05 ± 0.01	0.01 ± 0.00	0.10 ± 0.03	0.20 ± 0.05
Total tocopherols				0.12	0.26	0.28	0.03	0.05	0.01	0.10	0.20

KI = Kovats index, Rt = retention time.

**Table 3:** Relative percentage of volatile constituents in *P. mahaleb* seeds analyzed using SPME–GC–MS, *n* = 3. For codes, refer to Table 1.

No.	Volatiles	KI	Rt (min)	WG	WE	RE	RRE
**Organic and Fatty Acids**							
1	Formic acid	491.5	1.75	0.69 ± 0.42	1.01 ± 0.28	0.44 ± 0.06	1.31 ± 0.45
2	Acetic acid *	530.7	2.118	1.60 ± 0.32	2.23 ± 0.26	7.18 ± 2.21	1.27 ± 0.62
3	Propanoic acid	609.4	3.002	0.07 ± 0.06	0.02 ± 0.01	0.15 ± 0.07	0.04 ± 0.03
4	Butanoic acid	723	4.189	0.51 ± 0.31	0.50 ± 0.09	0.14 ± 0.08	0.24 ± 0.13
5	Valeric acid	873.4	5.796	9.22 ± 1.16	13.23 ± 3.73	6.09 ± 0.62	5.78 ± 2.04
6	Valeric acid isomer	901.8	6.046	9.22 ± 1.16	0.14 ± 0.12	0.04 ± 0.00	0.06 ± 0.01
7	Caproic acid	990.2	7.144	0.43 ± 0.04	0.24 ± 0.10	0.69 ± 0.20	0.82 ± 0.56
8	3-Hexenoic acid	1022.9	7.333	0.06 ± 0.01	0.05 ± 0.01	0.09 ± 0.07	0.18 ± 0.05
9	Heptanoic acid	1081.1	7.967	0.02 ± 0.01	0.02 ± 0.01	0.04 ± 0.01	0.09 ± 0.06
10	2-Ethylcaproic acid	1115.4	8.36	-	0.01 ± 0.00	0.05 ± 0.01	0.01 ± 0.00
11	2-Heptenoic acid	1113	8.393	0.13 ± 0.03	0.14 ± 0.02	0.21 ± 0.04	1.02 ± 0.74
12	Caprylic acid	1168	9.036	0.02 ± 0.00	0.02 ± 0.00	0.01 ± 0.00	0.63 ± 0.12
13	2-Octenoic acid	1205.2	9.275	0.13 ± 0.08	0.14 ± 0.03	0.35 ± 0.29	0.04 ± 0.03
14	Nonanoic acid	1267.7	9.911	0.01 ± 0.00	0.09 ± 0.03	0.21 ± 0.11	0.02 ± 0.00
15	Nonanoic acid isomer	1273	9.987	0.14 ± 0.03	0.43 ± 0.08	0.25 ± 0.02	1.10 ± 0.21
16	Capric acid	1347.8	10.686	0.04 ± 0.02	0.06 ± 0.00	0.06 ± 0.01	0.06 ± 0.00
17	Myristic acid	1759	14.679	0.02 ± 0.01	0.13 ± 0.09	0.22 ± 0.14	0.05 ± 0.02
18	Pentadecanoic acid	1848.8	15.6	0.04 ± 0.03	0.03 ± 0.02	0.07 ± 0.09	0.39 ± 0.55
19	Palmitoleic acid	1929.3	16.285	0.02 ± 0.01	0.09 ± 0.06	0.16 ± 0.13	0.03 ± 0.02
20	Palmitic acid	1949.3	16.481	0.11 ± 0.03	0.54 ± 0.37	0.96 ± 0.65	0.24 ± 0.10
Total acids				22.47	19.12	17.43	13.37
**Alcohols**							
21	1-Butanol	584.9	2.71	0.99 ± 0.57	0.04 ± 0.02	0.10 ± 0.03	0.02 ± 0.01
22	1-Pentanol (amyl alcohol)	704.8	4.021	1.91 ± 0.74	0.15 ± 0.07	0.52 ± 0.26	0.16 ± 0.07
23	2,3-Butanediol	721.2	4.167	0.12 ± 0.02	0.25 ± 0.19	0.98 ± 0.65	0.11 ± 0.08
24	2,3-Butanediol isomer	734.3	4.241	0.12 ± 0.02	0.25 ± 0.19	0.96 ± 0.66	0.18 ± 0.08
25	1-Hexanol*	837.7	5.401	0.47 ± 0.11	0.15 ± 0.11	1.45 ± 0.55	0.07 ± 0.02
26	1-Heptanol	960.7	6.725	0.36 ± 0.06	0.22 ± 0.11	0.50 ± 0.19	0.71 ± 0.25
27	3-Octenol	972.3	6.847	1.37 ± 0.24	1.07 ± 0.33	1.62 ± 0.81	0.97 ± 0.37
28	1-Octanol	1072.2	7.933	0.58 ± 0.10	0.91 ± 0.33	0.61 ± 0.13	3.20 ± 0.88
Total alcohols				5.93	3.05	6.75	5.44
**Aldehydes**							
29	Valeraldehyde	619.4	3.114	8.08 ± 3.24	0.51 ± 0.17	0.77 ± 0.31	0.29 ± 0.15
30	2-Pentenal	691.7	3.847	0.02 ± 0.01	0.02 ± 0.01	0.02 ± 0.01	0.01 ± 0.00
31	Caproaldehyde (Hexanal)	748.1	4.47	7.07 ± 2.20	0.36 ± 0.10	1.32 ± 0.51	0.80 ± 0.49
32	Nonanal *	817.3	5.221	0.12 ± 0.02	0.04 ± 0.02	0.11 ± 0.02	0.19 ± 0.23
33	Heptanal *	880	5.869	0.94 ± 0.15	2.44 ± 3.34	0.13 ± 0.02	0.72 ± 0.83
34	2,4-Heptadienal	916.7	6.243	0.28 ± 0.01	0.05 ± 0.02	0.10 ± 0.01	0.02 ± 0.01
35	2-Heptenal	947.3	6.574	0.66 ± 0.09	0.18 ± 0.05	0.51 ± 0.02	0.09 ± 0.03
36	Benzaldehyde *	955.4	6.658	0.88 ± 0.03	2.60 ± 0.94	18.71 ± 6.63	0.87 ± 0.61
37	Octanal	1001	7.192	0.07 ± 0.05	0.03 ± 0.01	0.06 ± 0.05	0.40 ± 0.24
38	Phenylacetaldehyde *	1049.9	7.668	0.06 ± 0.03	0.04 ± 0.01	0.20 ± 0.05	0.03 ± 0.01
39	2-Octenal	1061.4	7.785	1.05 ± 0.13	0.68 ± 0.24	1.52 ± 0.18	0.93 ± 0.36
40	Nonanal isomer	1108.7	8.302	1.48 ± 0.10	2.05 ± 0.90	3.24 ± 0.80	5.50 ± 2.54
41	2,4-Octadienal	1115.8	8.402	0.01 ± 0.00	0.02 ± 0.03	0.02 ± 0.01	0.01 ± 0.00
42	Decanal *	1123	8.448	0.02 ± 0.00	0.17 ± 0.04	1.16 ± 0.04	0.10 ± 0.01
43	2,4-Nonadienal	1133.4	8.661	0.05 ± 0.00	0.09 ± 0.02	0.11 ± 0.02	0.15 ± 0.01
44	2-Nonenal	1165.1	8.893	0.07 ± 0.01	0.10 ± 0.02	0.19 ± 0.12	0.13 ± 0.02
45	2-Propyl-2-heptenal	1190.9	9.176	0.24 ± 0.03	0.05 ± 0.01	0.14 ± 0.01	0.01 ± 0.00
46	2,4-Nonadienal isomer	1198.9	9.264	0.49 ± 0.16	0.82 ± 0.11	0.85 ± 0.01	0.19 ± 0.05
47	Cinnamaldehyde *	1222.3	9.471	7.25 ± 2.45	12.31 ± 1.67	11.19 ± 0.52	1.96 ± 0.34
48	4,5-Epoxy-2-decenal	1235.8	9.658	0.01 ± 0.00	0.01 ± 0.00	0.03 ± 0.02	0.38 ± 0.53
49	4-Oxononanal	1252.4	9.746	1.74 ± 0.44	3.95 ± 0.39	0.72 ± 0.07	1.96 ± 0.41
50	Cuminaldehyde *	1255.4	9.819	0.01 ± 0.00	0.24 ± 0.03	1.50 ± 0.39	0.04 ± 0.00
51	2-Decenal	1268.8	9.917	0.62 ± 0.06	1.00 ± 0.31	0.29 ± 0.07	3.64 ± 0.64
52	Cinnamaldehyde isomer	1285.4	10.078	4.88 ± 1.60	3.84 ± 1.29	0.21 ± 0.03	0.12 ± 0.01
53	2-Hydroxy-p-anisaldehyde	1345.3	10.668	0.32 ± 0.16	0.42 ± 0.21	0.09 ± 0.07	0.04 ± 0.01
54	Piperonal	1350.9	10.694	0.72 ± 0.63	0.34 ± 0.18	1.13 ± 0.39	0.54 ± 0.46
55	2-Undecenal	1369.7	10.758	0.03 ± 0.02	0.05 ± 0.01	0.09 ± 0.03	0.21 ± 0.16
56	Dodecanal	1396.4	11.232	0.15 ± 0.07	0.07 ± 0.01	0.13 ± 0.02	0.11 ± 0.09
57	Isovanillin (3-Hydroxy-p-anisaldehyde)	1409.3	11.256	0.02 ± 0.01	0.04 ± 0.01	0.07 ± 0.02	0.06 ± 0.05
Total aldehydes				37.34	32.52	44.60	19.48
**Aliphatic hydrocarbons**							
58	Dodecane	1112.9	8.341	0.17 ± 0.00	0.19 ± 0.01	0.08 ± 0.00	0.44 ± 0.06
59	Pentadecane	1482.7	11.96	0.05 ± 0.03	0.10 ± 0.02	0.01 ± 0.00	0.10 ± 0.00
60	2,2,4,6,6-Pentamethylheptane	1023.9	7.355	0.01 ± 0.00	0.01 ± 0.01	0.01 ± 0.00	0.01 ± 0.00
61	2,2,7,7-Tetramethyloctane	1031.8	7.492	0.07 ± 0.01	0.04 ± 0.01	0.11 ± 0.02	0.03 ± 0.02
62	Tetradecane *	1384.9	11.028	0.16 ± 0.07	0.13 ± 0.05	0.04 ± 0.01	0.04 ± 0.00
63	Hexadecane *	1599	13.199	0.01 ± 0.00	0.18 ± 0.06	0.23 ± 0.06	0.14 ± 0.13
64	Heptadecane *	1709.8	14.229	0.00 ± 0.01	0.02 ± 0.02	0.02 ± 0.01	0.12 ± 0.14
65	Octadecane *	1797.4	15.003	-	-	-	0.04 ± 0.05
66	Nonadecane *	1888.9	16.022	-	-	-	0.01 ± 0.01
Total aliphatic hydrocarbons				0.48	0.65	0.50	0.92
**Aromatics**							
67	Unknown	1326.2	10.473	2.46 ± 2.17	0.97 ± 0.54	3.32 ± 1.00	8.05 ± 8.15
68	Naphthalene *	1044.1	7.607	-	-	-	2.71 ± 3.83
69	Unknown	1207.9	9.335	0.10 ± 0.02	0.10 ± 0.03	0.05 ± 0.02	0.59 ± 0.17
Total aromatics				2.56	1.07	3.37	11.35
**Esters**							
70	Butyl formate	653.4	3.464	0.15 ± 0.07	0.01 ± 0.00	0.02 ± 0.00	-
71	*n*-Butyl acetate	768.2	4.524	0.87 ± 0.28	0.05 ± 0.01	0.13 ± 0.09	0.06 ± 0.04
72	Unknown	775.4	4.77	0.10 ± 0.04	0.13 ± 0.09	0.33 ± 0.11	0.13 ± 0.07
73	*n*-Pentyl formate	784.1	4.875	0.38 ± 0.13	0.02 ± 0.00	0.06 ± 0.01	0.01 ± 0.00
74	Hexyl formate	912	6.205	0.09 ± 0.02	0.03 ± 0.01	0.06 ± 0.01	0.02 ± 0.01
75	Benzyl formate	1084.6	8.13	0.03 ± 0.00	0.01 ± 0.00	0.09 ± 0.05	0.05 ± 0.03
76	Pentyl valerate	1096.3	8.168	1.65 ± 0.18	0.29 ± 0.19	1.22 ± 0.46	0.41 ± 0.42
77	Linalyl formate	1105	8.272	0.10 ± 0.00	0.08 ± 0.03	0.21 ± 0.03	0.07 ± 0.01
78	Octyl formate	1132.3	8.553	0.12 ± 0.01	0.19 ± 0.09	0.33 ± 0.08	0.69 ± 0.23
79	Octyl acetate	1137.9	8.678	0.11 ± 0.01	0.21 ± 0.05	0.28 ± 0.03	0.50 ± 0.12
80	Pentyl valerate isomer	1192	9.193	1.87 ± 0.16	0.81 ± 0.35	1.62 ± 0.30	0.62 ± 0.23
81	Octyl acetate isomer	1212.4	9.366	0.32 ± 0.07	0.79 ± 0.11	0.66 ± 0.05	3.26 ± 1.28
82	Pentyl caproate	1292.2	10.152	0.95 ± 0.28	0.61 ± 0.08	0.41 ± 0.02	2.25 ± 0.49
83	*α*-Terpinyl acetate *	1332.5	10.546	0.03 ± 0.01	0.03 ± 0.00	0.09 ± 0.03	0.03 ± 0.03
84	Hexyl caproate	1387.4	11.039	0.25 ± 0.11	0.24 ± 0.03	0.11 ± 0.01	0.27 ± 0.01
85	Benzyl valerate	1446.3	11.601	0.04 ± 0.02	0.05 ± 0.01	0.01 ± 0.00	0.06 ± 0.00
86	Octyl isovalerate	1476.2	11.949	0.06 ± 0.03	0.09 ± 0.02	0.03 ± 0.02	0.26 ± 0.01
87	Octyl hexanoate	1578.7	13.026	0.01 ± 0.00	0.01 ± 0.00	0.14 ± 0.09	0.05 ± 0.01
88	Benzyl Benzoate *	1783.5	14.903	0.02 ± 0.02	0.09 ± 0.09	0.09 ± 0.00	0.08 ± 0.02
89	Methyl palmitate	1915.6	16.137	0.01 ± 0.00	0.02 ± 0.01	0.05 ± 0.03	0.02 ± 0.02
Total esters				7.14	3.76	5.94	8.85
**Ethers**							
90	Estragole *	1207.4	9.319	0.01 ± 0.00	0.02 ± 0.00	0.05 ± 0.01	0.03 ± 0.03
91	Eugenol *	1342.7	10.584	-	-	-	0.08 ± 0.12
Total ethers				0.01	0.02	0.05	0.12
**Furans**							
92	2,3-Dihydrofuran	575.1	2.641	0.06 ± 0.01	0.03 ± 0.01	0.02 ± 0.01	0.01 ± 0.00
93	2-Propylfuran	736.5	4.345	0.05 ± 0.01	0.01 ± 0.01	0.02 ± 0.00	0.01 ± 0.00
94	3-Furfural *	794	4.955	0.01 ± 0.01	0.01 ± 0.00	0.05 ± 0.03	0.01 ± 0.01
95	2-Butylfuran	868.9	5.737	1.49 ± 0.07	0.27 ± 0.13	0.49 ± 0.12	0.15 ± 0.08
96	2-Pentylfuran	988.5	7.012	2.65 ± 0.23	0.73 ± 0.30	2.73 ± 0.71	1.77 ± 0.92
97	4-Methyl-2-propyl furan	1033.9	7.538	0.18 ± 0.03	0.04 ± 0.02	0.12 ± 0.02	0.06 ± 0.03
98	5-Methyl-2-propionylfuran	1152.1	8.726	0.21 ± 0.02	0.22 ± 0.04	0.01 ± 0.00	0.06 ± 0.02
99	1-(2-Furyl)-3-butene-1,2-diol	1174.1	8.959	0.74 ± 0.11	1.10 ± 0.05	0.39 ± 0.04	4.30 ± 0.99
Total furans				5.41	2.39	3.84	6.37
**Ketones**							
100	2-Hexanone	735.5	4.327	0.14 ± 0.03	0.09 ± 0.10	0.95 ± 0.53	0.06 ± 0.03
101	3-Methyl-1-cyclopentanone	812.3	4.956	0.04 ± 0.01	0.05 ± 0.03	0.13 ± 0.10	0.05 ± 0.02
102	3-Octen-2-one	1040.8	7.568	2.31 ± 0.29	0.82 ± 0.33	2.88 ± 0.73	1.21 ± 0.56
103	5-Decanone	1057.5	7.68	0.44 ± 0.05	0.30 ± 0.08	0.39 ± 0.16	0.07 ± 0.01
104	5-Nonanone	1076.7	7.956	0.28 ± 0.02	0.07 ± 0.02	0.22 ± 0.04	0.04 ± 0.01
105	2-Nonanone	1095.9	8.143	0.02 ± 0.00	0.03 ± 0.01	0.16 ± 0.01	0.02 ± 0.00
106	5-Decanone isomer	1176.3	9.06	0.05 ± 0.00	0.05 ± 0.00	0.02 ± 0.00	0.05 ± 0.02
107	2-Decanone	1195.5	9.231	0.04 ± 0.00	0.12 ± 0.03	0.16 ± 0.01	0.90 ± 0.34
108	Cyclononanone	1249.5	9.666	0.01 ± 0.01	0.02 ± 0.01	0.05 ± 0.01	0.04 ± 0.04
109	Carvone *	1258.1	9.831	-	-	-	0.02 ± 0.03
110	6-Undecanone	1277.6	10.013	0.30 ± 0.08	0.30 ± 0.05	0.25 ± 0.03	0.76 ± 0.22
111	6-Dodecanone	1375.5	10.928	0.13 ± 0.06	0.63 ± 0.17	0.16 ± 0.03	0.48 ± 0.08
Total ketones				3.77	2.50	5.37	3.69
**Lactones**							
112	*γ*-Butyrolactone	897	6.025	1.01 ± 0.17	1.43 ± 1.98	0.08 ± 0.01	0.02 ± 0.00
113	*γ*-Pentalactone	945.1	6.548	0.51 ± 0.09	0.59 ± 0.22	0.53 ± 0.11	0.27 ± 0.13
114	*γ*-Caprolactone	1059.9	7.775	1.14 ± 0.16	2.10 ± 0.77	2.14 ± 0.42	6.58 ± 2.38
115	*γ*-Heptalactone	1161.8	8.87	0.09 ± 0.01	0.26 ± 0.07	0.19 ± 0.01	0.86 ± 0.27
116	*γ*-Octalactone	1269.5	9.931	0.82 ± 0.21	1.33 ± 0.14	0.36 ± 0.04	2.77 ± 1.07
117	*δ*-Octalactone	1299.3	10.253	0.05 ± 0.01	0.06 ± 0.01	0.14 ± 0.06	0.14 ± 0.04
118	Isomintlactone	1356.2	10.736	1.74 ± 0.68	4.34 ± 1.03	1.19 ± 0.24	8.31 ± 1.60
119	*γ*-Nonalactone	1373.6	10.921	0.76 ± 0.30	2.49 ± 0.26	0.88 ± 0.17	4.20 ± 0.55
120	*δ*-Nonalactone	1402.5	11.246	0.03 ± 0.02	0.02 ± 0.00	0.13 ± 0.05	0.23 ± 0.30
121	Coumarin isomer	1433.8	11.518	0.04 ± 0.02	0.23 ± 0.04	0.02 ± 0.01	0.03 ± 0.03
122	Coumarin *	1451.3	11.702	7.28 ± 3.33	19.75 ± 8.56	1.14 ± 0.30	0.78 ± 0.18
123	Herniarin	1750.7	14.616	0.78 ± 0.29	1.37 ± 0.88	0.04 ± 0.01	0.07 ± 0.02
Total lactones				14.24	33.99	6.84	24.25
**Monoterpenes**							
124	Limonene *	1033.6	7.529	0.17 ± 0.02	0.48 ± 0.11	4.35 ± 0.09	0.07 ± 0.03
125	*p*-Cymene *	1028.7	7.435	0.12 ± 0.02	0.06 ± 0.03	0.23 ± 0.06	0.16 ± 0.06
126	Thymol *	1309.1	10.313	0.01 ± 0.00	0.01 ± 0.00	0.06 ± 0.04	0.24 ± 0.06
127	*α*-Terpineol *	1084.2	8.106	0.02 ± 0.00	0.02 ± 0.01	0.10 ± 0.02	0.09 ± 0.02
Total monoterpenes				0.31	0.57	4.74	0.57
**Sesquiterpenes**							
128	Cadalene	1706	14.127	-	-	0.08 ± 0.07	0.03 ± 0.03
129	*α*-Copaene	1395	11.223	0.10 ± 0.09	0.05 ± 0.02	0.11 ± 0.04	0.16 ± 0.13
130	Longifolene	1428.1	11.462	0.01 ± 0.01	0.03 ± 0.00	0.06 ± 0.01	0.04 ± 0.01
131	*α*-Muurolene	1507.2	12.269	0.01 ± 0.00	0.08 ± 0.03	0.11 ± 0.05	0.01 ± 0.00
132	*β*-Caryophyllene *	1437.2	11.537	0.01 ± 0.01	0.02 ± 0.00	0.04 ± 0.01	0.05 ± 0.01
Total sesquiterpenes				0.12	0.18	0.39	0.28
**Unidentified volatiles**							
133	Unknown	1305.4	10.393	0.01 ± 0.00	0.02 ± 0.00	0.01 ± 0.00	0.08 ± 0.10
134	Unknown	1667.4	13.841	0.01 ± 0.01	0.02 ± 0.02	0.07 ± 0.09	4.98 ± 7.03
135	Unknown	1821.9	15.264	0.18 ± 0.13	0.13 ± 0.09	0.09 ± 0.04	0.27 ± 0.29
Total unidentified volatiles				0.21	0.18	0.17	5.33

KI = Kovats index, Rt = retention time. * Denotes volatiles confirmed using authentic standards.

## Supplementary Materials

The following are available online at https://www.mdpi.com/article/10.3390/foods10040728/s1, Figure S1: Representative SPME–GC–MS chromatograms of volatiles, collected from the extracts of (a) white mahlab from Greece “WG”, (b) white mahlab from Egypt “WE”, (c) red mahlab from Egypt “RE” and (d) roasted red mahlab from Egypt “RRE”. Figure S2: GC–MS based OPLS-DA score plot (a) derived from modeling aroma profile of unroasted red RE *versus* red roasted RRE mahaleb (*n* = 3). The respective loading S-plots (b) showing the covariance p [1] against the correlation p(cor) [1] of the variables of the discriminating component of the OPLS-DA model. Designated variables are highlighted and identifications are discussed in the text.
