# Supplementary material for: Profiling of Primary Metabolites and Volatile Determinants in Mahlab Cherry (Prunus mahaleb L.) Seeds in the Context of Its Different Varieties and Roasting as Analyzed Using Chemometric Tools
Source: Foods. 2021 Mar 30;10(4):728. doi: 10.3390/foods10040728 (PMC8066072; doi:10.3390/foods10040728)
Supplement: Supplementary file 1 [file foods-10-00728-s001.pdf]

Supplemental Material

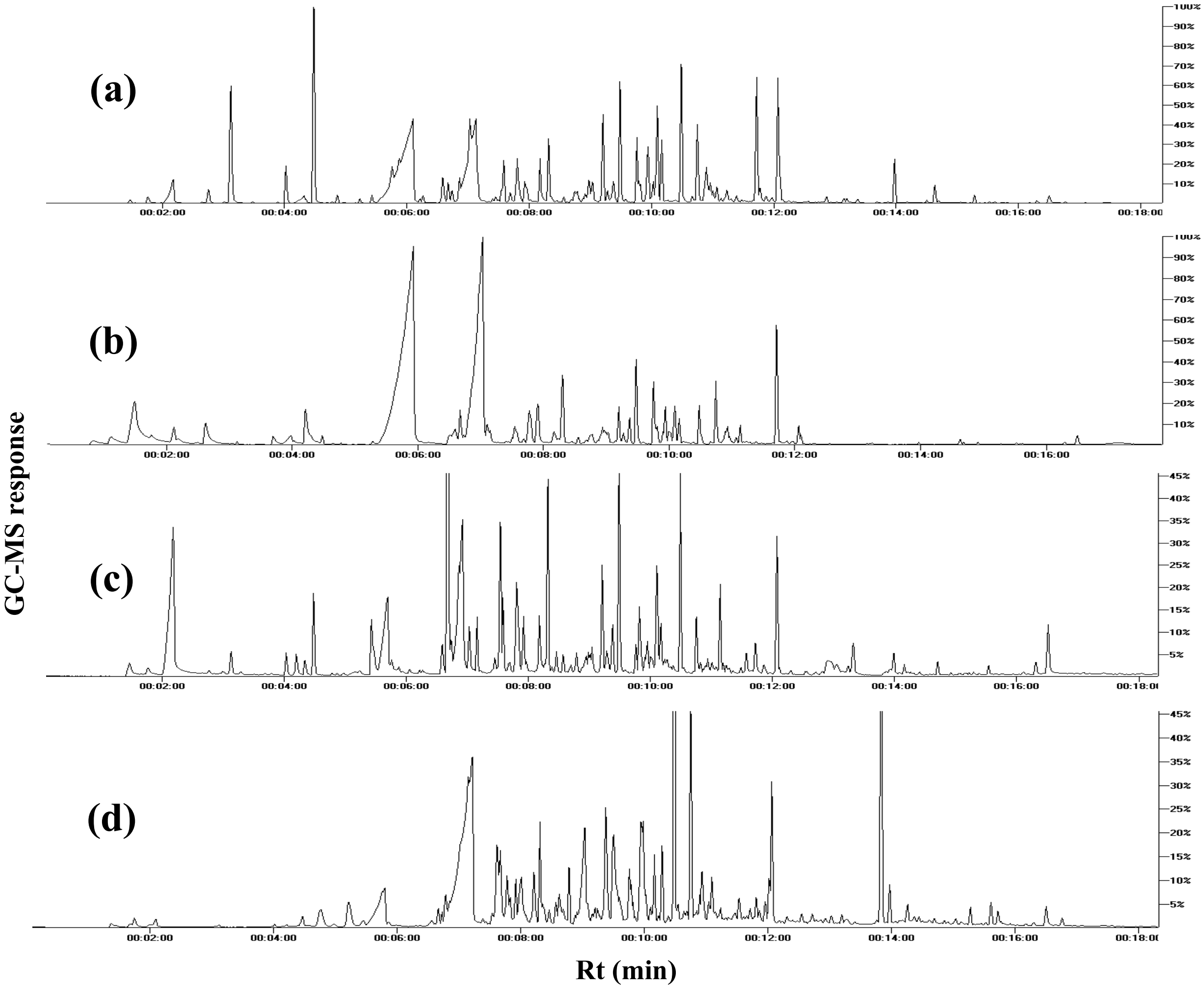

Figure S1. Representative SPME-GC–MS chromatograms of volatiles, collected from the extracts of (a) white mahlab from Greece "WG", (b) white mahlab from Egypt "WE", (c) red mahlab from Egypt "RE" and (d) roasted red mahlab from Egypt "RRE".

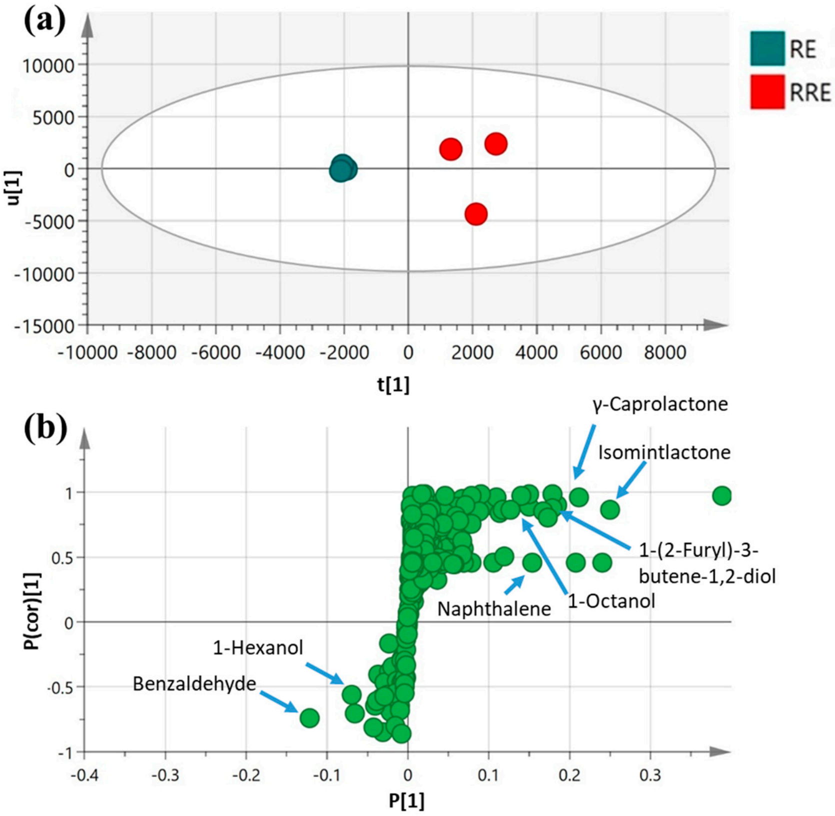

Figure S2. GC-MS based OPLS-DA score plot (a) derived from modelling aroma profile of unroasted red RE versus red roasted RRE mahaleb (n = 3). The respective loading S-plots (b) showing the covariance p[1] against the correlation p(cor)[1] of the variables of the discriminating component of the OPLS-DA model. Designated variables are highlighted and identifications are discussed in the text.
